# A Voltage-Gated H^+^ Channel Underlying pH Homeostasis in Calcifying Coccolithophores

**DOI:** 10.1371/journal.pbio.1001085

**Published:** 2011-06-21

**Authors:** Alison R. Taylor, Abdul Chrachri, Glen Wheeler, Helen Goddard, Colin Brownlee

**Affiliations:** 1 The Marine Biological Association, The Laboratory, Citadel Hill, Plymouth, United Kingdom; 2 Department of Biology and Marine Biology, University of North Carolina Wilmington, Wilmington, North Carolina, United States of America; 3 Plymouth Marine Laboratory, Prospect Place, The Hoe, Plymouth, United Kingdom; Rutgers The State University of New Jersey, United States of America

## Abstract

Marine coccolithophorid phytoplankton are major producers of biogenic calcite, playing a significant role in the global carbon cycle. Predicting the impacts of ocean acidification on coccolithophore calcification has received much recent attention and requires improved knowledge of cellular calcification mechanisms. Uniquely amongst calcifying organisms, coccolithophores produce calcified scales (coccoliths) in an intracellular compartment and secrete them to the cell surface, requiring large transcellular ionic fluxes to support calcification. In particular, intracellular calcite precipitation using HCO_3_^−^ as the substrate generates equimolar quantities of H^+^ that must be rapidly removed to prevent cytoplasmic acidification. We have used electrophysiological approaches to identify a plasma membrane voltage-gated H^+^ conductance in *Coccolithus pelagicus ssp braarudii* with remarkably similar biophysical and functional properties to those found in metazoans. We show that both *C. pelagicus* and *Emiliania huxleyi* possess homologues of metazoan H_v_1 H^+^ channels, which function as voltage-gated H^+^ channels when expressed in heterologous systems. Homologues of the coccolithophore H^+^ channels were also identified in a diversity of eukaryotes, suggesting a wide range of cellular roles for the H_v_1 class of proteins. Using single cell imaging, we demonstrate that the coccolithophore H^+^ conductance mediates rapid H^+^ efflux and plays an important role in pH homeostasis in calcifying cells. The results demonstrate a novel cellular role for voltage gated H^+^ channels and provide mechanistic insight into biomineralisation by establishing a direct link between pH homeostasis and calcification. As the coccolithophore H^+^ conductance is dependent on the trans-membrane H^+^ electrochemical gradient, this mechanism will be directly impacted by, and may underlie adaptation to, ocean acidification. The presence of this H^+^ efflux pathway suggests that there is no obligate use of H^+^ derived from calcification for intracellular CO_2_ generation. Furthermore, the presence of H_v_1 class ion channels in a wide range of extant eukaryote groups indicates they evolved in an early common ancestor.

## Author Summary

The production of calcium carbonate structures by marine organisms has a major influence on the Earth's carbon cycle and is responsible for the eventual formation of sedimentary rocks such as chalk and limestone. The major contributors to marine calcification are the coccolithophores, a family of unicellular algae which surround themselves in calcified plates known as coccoliths. Unlike many other calcifying organisms, coccolithophores produce their calcified structures inside the cell, enabling precise control of this process. However, the other product resulting from the calcification reaction, H^+^, must be rapidly removed to maintain the pH inside the cell. In this study, we show that coccolithophores possess a voltage-gated H^+^ channel, which removes H^+^ rapidly from the cell during calcification and helps maintain a constant pH. We identify the gene encoding this H^+^ channel, HVCN1, and find that it is a distant relative of those recently identified in animal cells, suggesting that H^+^ channels may be present in many other types of eukaryote organism. As calcifying organisms may be affected by ocean acidification, the identification of an H^+^ channel in coccolithophores gives us an important mechanistic understanding of cellular pH regulation during the calcification process, and may give insight into the response of coccolithophores to future changes in ocean pH.

## Introduction

Coccolithophores represent a pan-global group of oceanic phytoplankton, often forming massive monospecific blooms in oceanic waters. These unicellular eukaryote algae produce highly intricate calcium carbonate scales, known as coccoliths, and are the most numerous calcifying organisms in our oceans. Globally abundant species such as *Emiliania huxleyi* and *Coccolithus pelagicus spp braarudii* [1] play fundamental roles in long-term carbon deposition, marine biogeochemical cycling, and atmospheric chemistry through their direct effects on surface ocean alkalinity and through ballasting of organic carbon fluxes to deeper waters [2]. Anthropogenic CO_2_ emissions are predicted to have a significant impact on calcifying organisms, due to a decrease in both ocean surface water pH and the saturation state of calcium carbonate. However, there is currently significant debate regarding the effects of elevated CO_2_ and decreased ocean pH on coccolithophores [3]–[7], due in part to a lack of understanding of the cellular mechanisms underlying calcification. Improved knowledge of coccolithophore cell biology is therefore necessary for both predicting the physiological consequences of ocean acidification and identifying experimental versus physiological sources of variability observed in experimental manipulations on this ubiquitous group of phytoplankton.

In contrast to other major marine calcifyers such as corals [8] and foraminifera [9], coccolithophores produce calcite scales (heterococcoliths) entirely within intracellular compartments (the coccolith vacuole, CV) which are secreted to the cell surface forming the external coccosphere [10]. Intracellular calcification requires large sustained fluxes of Ca^2+^ and inorganic carbon (C_i_) to the coccolith vacuole. The bulk of experimental work supports HCO_3_^−^ as the primary C_i_ species transported into the cell to sustain calcification, resulting in 1 mole of H^+^ generated for every 1 mole of calcite precipitated [11],[12]. Our calculations, based on published calcification rates, indicate that H^+^ production during calcification without H^+^ removal or consumption will cause rapid cytoplasmic acidification of ∼0.3 pH min^−1^ (Table S1). Metabolic pH balance may arise through photosynthesis [13], though the degree to which this occurs has been questioned by studies that indicate no mechanistic dependence of photosynthesis on calcification [14],[15]. It follows that in the absence of rapid metabolic H^+^ consumption, sequestration, or removal, the cytosol of calcifying coccolithophore cells would be subject to significant acidosis. Our data show that a plasma membrane voltage-activated H^+^ channel, novel for photosynthetic organisms, plays a crucial role in short-term cellular pH homeostasis which is in turn required for maintenance of calcification.

## Results

**Figure 1:**
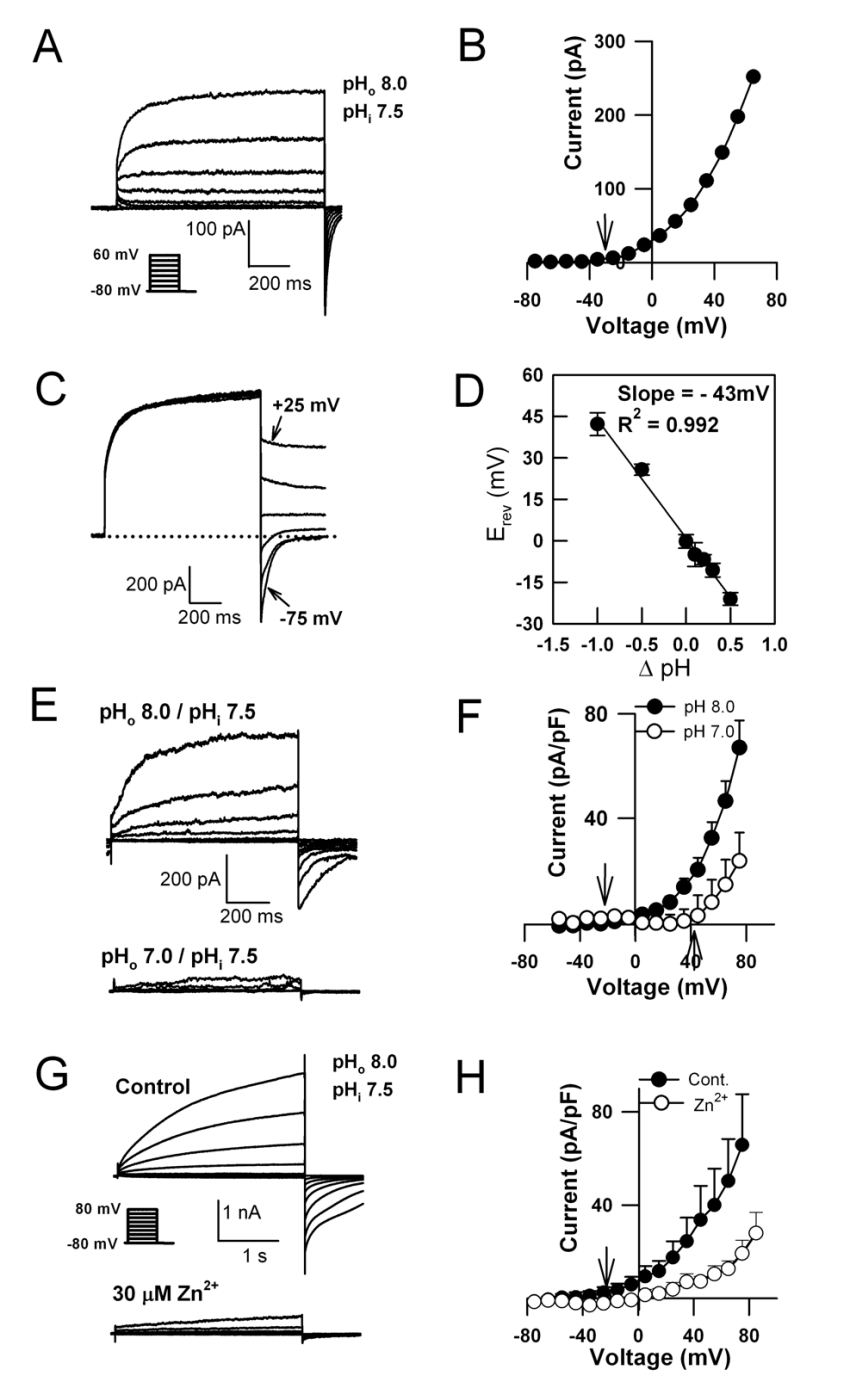
Biophysical characteristics of the H^+^ current in *C. pelagicus*. (A) Whole cell currents from *C. pelagicus* cells in response to incremental 1 s 10 mV depolarisations from −80 to +60 mV. The recording pipette contained (in mM) K-Glutamate 200, MgCl_2_ 5, EGTA 5, and HEPES 100 (P1a, Table S2). The ASW bathing solution contained (in mM) NaCl 450, KCl 8, MgCl_2_ 30, MgSO_4_ 16, CaCl_2_ 10, NaHCO_3_ 2, and HEPES 20 (E1, Table S2). For clarity only every other trace (Δ+20 mV) is indicated. (B) Corresponding current-voltage relationship showing outward current activation at voltages more positive than E_H_^+^ (arrow). (C) Tail currents activated by a 1 s depolarizing pulse to +40 mV before 500 ms test pulses from −75 to +25 mV. The dotted line indicates zero current. Solutions are as in (A). (D) pH gradient (ΔpH  =  pH_o_ − pH_i_) dependence of average E_rev_ (± SE, *n* = 6). Data were fitted by a regression with a slope of −43 mV/pH unit. (E) pH_o_ sensitivity of whole current activation. The pipette solution contained (in mM): MgCl_2_ 5, EGTA 5, TEA-Cl 200, and HEPES 5 (P2, Table S2), and the ASW bath solution was as in (A). (F) Average whole cell currents (± SE) under the same conditions as (E) in pH_o_ 8.0 (*n* = 10) and 7.0 (*n* = 4). Outward currents are smaller and activation more positive with decreased pH_o_ (arrows  =  E_H_^+^). (G) Effect of 30 µM free Zn^2+^ on the outward currents. The internal and external solutions used are the same as in (A) (P1a, E1). (H) Average current voltage curves (± SE) for control (*n* = 5) and in the presence of 30 µM free Zn^2+^ (*n* = 5). Arrow indicates E_H_^+^.

In order to understand the membrane transport processes which underlie the extraordinary process of intracellular calcification in coccolithophores, we applied the patch clamp technique following removal of the external calcite coccolith scales by brief treatment with the Ca^2+^ chelator ethyleneglycol-O, O'-bis(2-aminoethyl)-N, N, N', N'-tetraacetic acid (EGTA) (see Materials and Methods; Figure S1). Patch clamp recordings revealed a slowly activating plasma membrane ion current in *C. pelagicus* in response to depolarisations more positive than the equilibrium potential for H^+^ (E_H_^+^) (Figures 1A,B). Tail current analysis demonstrated a reversal potential (E_rev_) very positive of E_K_^+^ and E_Cl_^−^, and closest to E_H_^+^ (Figures 1C,D). A strong Nernstian relationship between E_rev_ and transmembrane pH gradient (ΔpH) in the presence of various bath (pH_ο_ 6.5–8.0) solutions showed that the current is selective for H^+^ (Figure 1D). The outward current was depressed and voltage activation shifted more positive in response to decreased pH_ο_ (Figure 1E,F), a characteristic of animal H^+^ currents [16],[17]. Reducing pH_i_ from 7.5 to 6.5 (with pH_o_ held at 8.0) resulted in a greater outward current amplitude at all membrane potentials (Figure S2), although it was not possible to determine accurately the activation potential of the H^+^ current at pH_i_ 6.5 as E_H_^+^ was too close to the activation potential for the inward Cl^−^ current [18].

**Figure 2:**
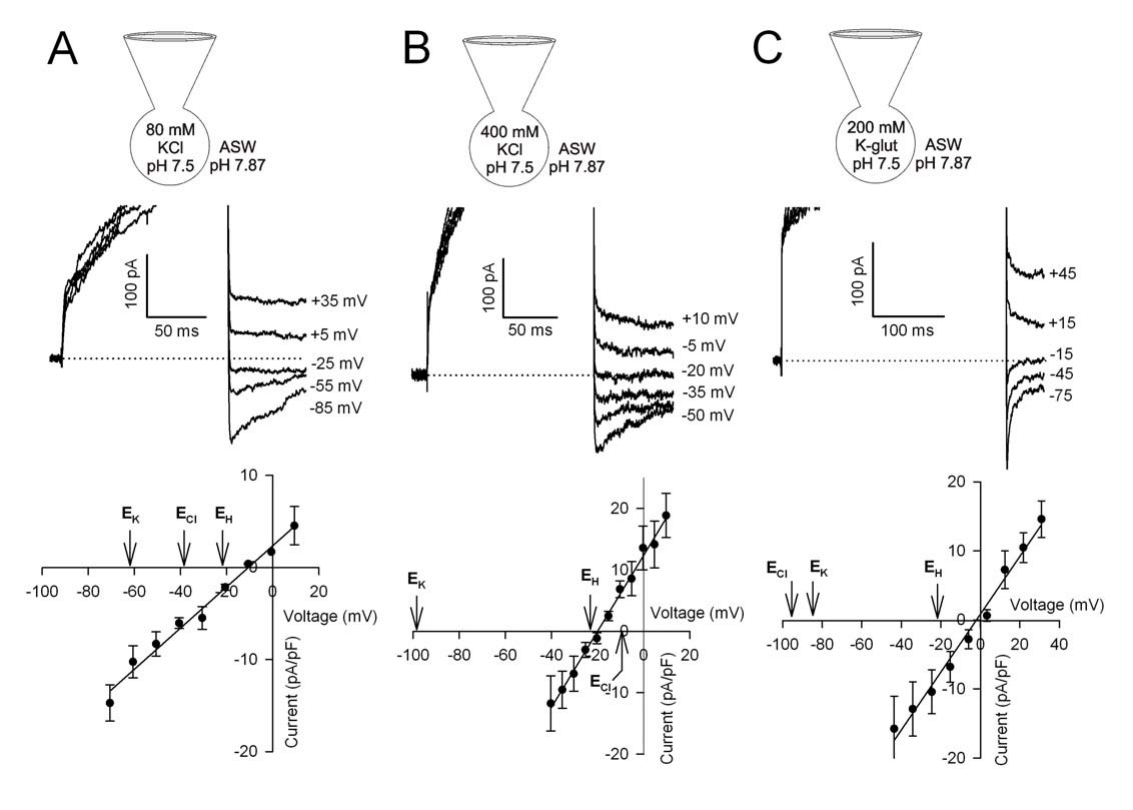
*C. pelagicus* H^+^ currents are insensitive to changes in [K^+^] and [Cl^−^]. Tail current analysis was conducted with a range of pipette solutions with major salt as indicated in the schematic KCl 80 mM (A), KCl 400 mM (B), and K-Glutamate 200 mM (C) and 5 mM HEPES buffer. All other constituents were as P1 (Table S2), which are (in mM) MgCl_2_ 5, EGTA 5. The bath solution was ASW containing (in mM) NaCl 450, KCl 8, MgCl_2_ 30, MgSO_4_ 16, CaCl_2_ 10, NaHCO_3_ 2, and HEPES 20; and adjusted to the pH indicated in each schematic (E1, Table S2). Pipette solutions were adjusted to ∼1,000 mOsmol kg^−1^ with sorbitol. For each recording condition, outward currents were activated by a depolarising voltage pulse to +110 mV and tail currents subsequently recorded at a range of voltages. The upper panels show representative tail current traces for (A) 80 mM KCl, (B) 400 KCl, and (C) 200 K-glutamate pipette solutions. Dotted lines represent zero current and voltages for representative traces are indicated. The initial activated outward currents are truncated to emphasise the tail currents. Lower plots illustrate average tail current-voltage plots for a minimum of 4 cells for each condition (±SE). Arrows indicate the predicted reversal potential for each of the major ions. The tail current reversal for the *C. pelagicus* outward currents are consistently close to E_H_^+^ regardless of E_Cl_^−^ and E_K_^+^, suggesting whole cell currents activated by depolarisation are primarily carried by H^+^ ions in calcifying cells. The slightly positive reversal indicated may be due in part to the weakly buffered pipette solution used in these experiments or because of a minor contribution by endogenous cation currents carried by Mg^2+^ Ca^2+^ or Na^+^ (predicted equilibrium potentials of +26, +182, and > +300 mV for each ion, respectively).

*C. pelagicus* H^+^ currents were inhibited by Zn^2+^ (Figure 1G,H), which is characteristic of animal H^+^ currents [19],[20] and were sensitive to the trivalent cation Gd^3+^ (Figure S3). To confirm that K^+^ or Cl^−^ was not contributing significantly to the outward current, tail current analysis was performed using a range of pipette solutions. In all cases the reversal potentials of *C. pelagicus* outward currents were consistently close to E_H_^+^ regardless of large changes in E_K_^+^ or E_Cl_^−^ (Figure 2). The small deviation observed from E_H_^+^ may be due to incomplete pH buffering by the pipette solution [21],[22] or a small contribution from an additional unidentified conductance. Application of the Goldman, Hodgkin, Katz equation for relative permeabilities of the ions in the pipette solutions (H^+^, K^+^, and Cl^−^) from the data in Figures 1 and 2 gives permeability ratios in excess of 10^6^ for gH^+^ relative to K^+^ and Cl^−^. The extremely high selectivity for H^+^ is consistent with other reported values for H^+^ channels (e.g., [16]). The observed H^+^ current magnitudes in *C. pelagicus* are comparable to the large currents found in activated granulocytes [21] and more than adequate to dissipate calcification associated H^+^ production in the absence of metabolic consumption or sequestration (Table S1). While a range of H^+^ transport and homeostatic mechanisms are likely to contribute to pH_i_ regulation, the H^+^ efflux channel identified here possesses the transport capacity and kinetics that would enable rapid short-term regulation of potentially large pH_i_ fluctuations.

**Figure 3:**
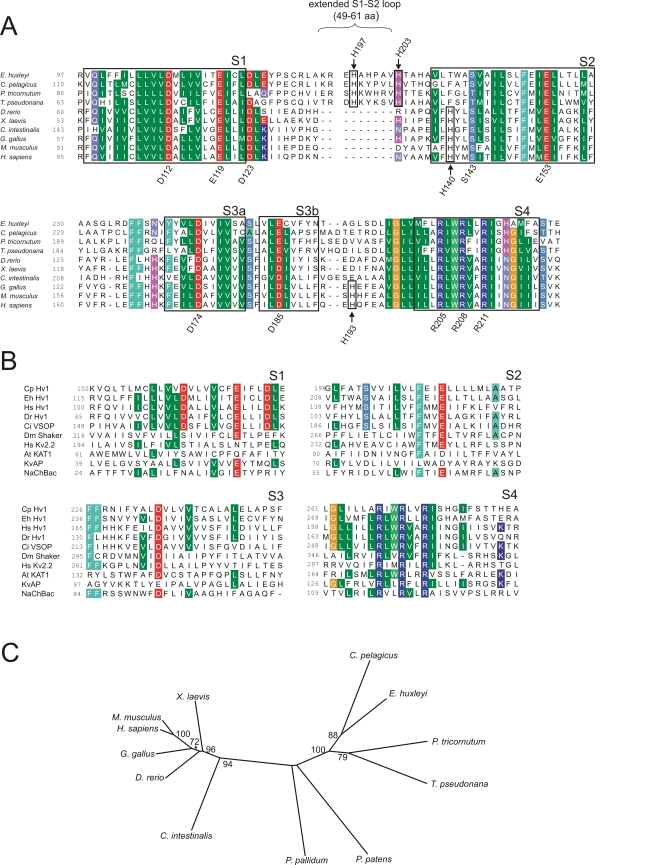
Conservation of amino acid sequences between H_v_1 orthologues. (A) Multiple sequence alignment of animal and coccolithophore H_v_1 proteins. The multiple sequence alignment indicates transmembrane residues conserved between animal and coccolithophore H_v_1 proteins. Shading indicates residues that are identical or similar in 80% of the sequences (BLOSUM62 matrix), and boxes represent predicted transmembrane domains in human H_v_1. The three arginine residues in S4 required for voltage sensing are conserved (numbers below alignment correspond to R205, R208, and R211 in human H_v_1). Many of the acidic residues (aspartate and glutamate) appear conserved (D112, E119, D123, E153, D174), although Asp185 is replaced by glutamate and Glu171 is not conserved. Several of these acidic residues are conserved amongst voltage sensor domain proteins and may function in voltage sensing [29]. In contrast, the other basic residues (lysine) are not conserved in coccolithophores (K125, K157, K169, K221). Polar serine residues (S143, S181) are conserved in *E. huxleyi*, although Ser181 is replaced by alanine in *C. pelagicus*. Similar sequences identified in the diatoms *P. tricornutum* and *T. pseudonana* are also shown in the alignment. The arrows indicate external histidine residues required for Zn^2+^ inhibition in human H_v_1 [19] and external histidine residues which are conserved in algal H_v_1 proteins (the numbers above the alignment indicate their position in EhH_v_1). (B) Multiple sequence alignment indicating conserved domains in transmembrane regions of voltage sensor proteins. H_v_1 proteins from animals (Hs, *Homo sapiens*; Dr, *Danio rerio*; Ci, *Ciona intestinalis*) and coccolithophores (Eh, *Emiliania huxleyi*; Cp, *Coccolithus pelagicus*) are aligned to transmembrane regions S1–S4 from K^+^ channels from humans, *Drosophila*, plants, and prokaryotes and also to the prokaryote Na^+^ channel NaChBac. Shading indicates identical residues in 5 out of 10 sequences displayed. In this analysis the only conserved residues that are exclusive to H_v_1 proteins correspond to human H_v_1 residues D112, D123, and S143. (C) Phylogenetic analysis of known H_v_1 proteins. The tree was generated by a maximum likelihood analysis of available H_v_1 protein sequences. 100 bootstraps were performed.

The outward H^+^ conductance in *C. pelagicus* shares many characteristics with those produced by the H_v_1 class of voltage-gated H^+^ channels identified in animals [17],[19],[20]. Similarity searches using animal H_v_1 sequences identified a single putative open reading frame coding for 339 amino acids (*EhHVCN1*) within the *E. huxleyi* genome (Joint Genome Institute; Read et al., unpublished). We subsequently identified a further putative homologue in a collection of *C. pelagicus* ESTs (*CpHVCN1*, von Dassow et al., unpublished). The coccolithophore sequences exhibit a weak overall similarity to mammalian H_v_1 channels at the amino acid level (EhH_v_1 has 19% identity, 33% similarity to human H_v_1), but have similar organisation including four predicted transmembrane domains and conserved features including the critical voltage sensing arginine residues in transmembrane domain S4 (Figure 3A–C). Notable differences are the non-conservation of histidine residues required for Zn^2+^ inhibition in mammalian H_v_1 channels [19] and extension of the putative extracellular loop between the S1 and S2 domains. RT-PCR confirmed that *EhHVCN1* and *CpHVCN1* were expressed in calcifying strains of *E. huxleyi* and *C. pelagicus*, respectively. Sequence similarity searches of currently available genomic datasets using the coccolithophore proteins identified further putative H_v_1 homologues in several evolutionarily distant eukaryotes including the diatoms, *Phaeodactylum tricornutum* and *Thalassiosira pseudonana*, and the social amoebozoan, *Polysphondylium pallidum*, indicating that the H_v_1 class of proteins may have a broad taxonomic distribution and an ancient evolutionary origin (Figure 3A,C).

**Figure 4:**
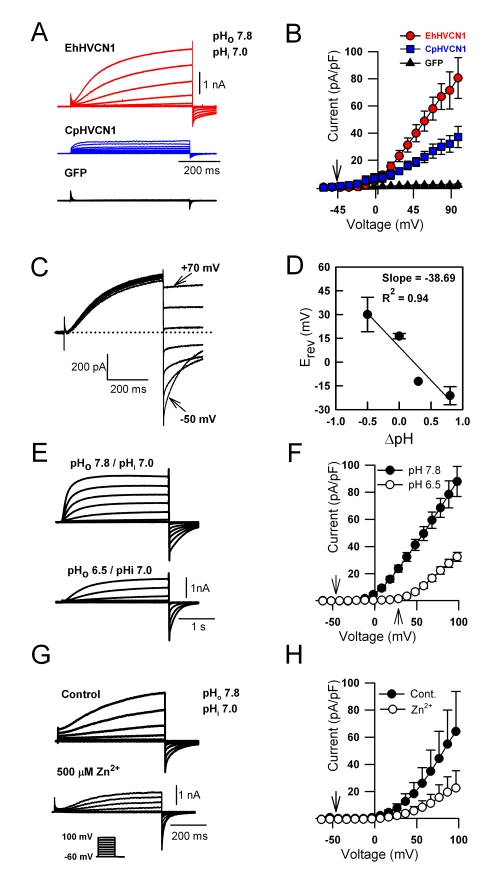
H_v_1 homologues in coccolithophores function as H^+^ channels. (A) Whole cell currents in HEK293 cells transfected with *EhHVCN1*, *CpHVCN1*, and *GFP* only. The pipette solution contained (in mM) NMDG 65, MgCl_2_ 3, EGTA 1, and HEPES 150 glucose 70 at pH 7.0 (P4, Table S2), and the bath solution contained (in mM) N-methyl D-glucamine (NMDG) 75, MgCl_2_ 3, CaCl_2_ 1, glucose 160, and HEPES 100 at pH 7.8 (E4, Table S2). Cells were depolarised in 10 mV increments from −60 to +100 mV. For clarity, every other trace is shown. (B) Average current-voltage curves (± SE, *n* = 6) of HEK293 cells transfected with *EhHVCN1* (red circles), *CpHVCN1* (blue squares), and *GFP* only (black triangles), respectively, using the protocol described in (A). (C) Tail current analysis of HEK293 cells expressing *EhHVCN1*. Currents were activated by a 500 ms depolarisation to +80 mV followed by repolarisation to between −50 and +70 mV. Dotted line represents zero current level. The solutions used for patch and bath solutions were the same as described in (A) (E4, P4). (D) pH dependence of EhH_v_1 E_rev_. (E) Outward currents of EhH_v_1-expressing HEK 293 cells activated by depolarising pulses from −60 mV to +100 mV before (upper panel) and after (lower panel) perfusion with pH_o_ 6.5. The pipette solution was as in (A) and the external solutions were as described above except that 100 mM MES replaced 100 mM HEPES at pH 6.5 (E5, Table S2). (F) Average current-voltage curves (± SE, *n* = 5) for pH_o_ 7.8 or 6.5. Activation voltage shifts with E_H_^+^ (arrows). (G) Inhibition of EhH_v_1 currents by 500 µM Zn^2+^. The recording pipette contained in mM: NaCl 30, KCL 100, MgCl_2_ 3, EGTA 1, and HEPES 100 at pH 7.0 (P3, Table S2). Cells were bathed with (in mM) NaCl 160, KCL 2.0, MgCl_2_ 1, CaCl_2_ 1, Glucose 20, and HEPES 100 at pH 7.8 (E3, Table S2). (H) Average current voltage curves (± SE, *n* = 5) for control and in the presence of 500 µM Zn^2+^.

Human HEK293 cells transfected with either *EhHVCN1* or *CpHVCN1* exhibited robust voltage-dependent outward currents significantly greater than endogenous outward currents known to occur in this cell type (Figure 4A,B) [17]. Further characterisation of EhH_v_1 expressed in HEK293 cells indicated that the magnitude and E_rev_ of the current was pH dependent (Figure 4C–F) and sensitive to Zn^2+^ (Figure 4G,H). Analysis of H^+^ current activation kinetics in response to +50 mV depolarisation shows that the currents generated by heterologously expressed EhH_v_1 and CpH_v_1 had faster activation kinetics than the *C. pelagicus* native currents (τ  = 107 ms ±30.4 SE, 22.9 ms ±5.4 SE, and 220 ms ±39.3 SE for EhH_v_1, CpH_v_1 in HEK cells, and *C. pelagicus* native conductances, respectively). This probably reflects the different cellular context of the native and HEK cell expression since it is well documented that activation time constant can be strongly influenced by physiological conditions [23].

As the external histidine residues associated with Zn^2+^ binding in human H_v_1 (H140, H193) are not conserved in coccolithophore H_v_1 proteins, we hypothesised that Zn^2+^ must bind alternative residues in order to inhibit the H^+^ conductances in these channels. We identified two histidine residues in the predicted S1–S2 extracellular loop region which are completely conserved across the available coccolithophore and diatom H_v_1 sequences and used site-directed mutagenesis of EhH_v_1 to examine whether they contributed to inhibition by Zn^2+^. Replacement of either histidine residue (H197 or H203) with an alanine residue resulted in a very substantial reduction in the extent of inhibition induced by 500 µM Zn^2+^ (from 84% to 27% or 28%, respectively, Figure S4), suggesting that whilst the individual histidine residues concerned are not conserved between animal and algal H_v_1 proteins, the mechanism of inhibition by Zn^2+^ may be similar. We conclude that coccolithophores express functional homologues of mammalian voltage gated H^+^ channels which exhibit highly similar biophysical properties to the outward H^+^ conductance observed in *C. pelagicus*, strongly suggesting that this conductance is generated by CpH_v_1.

**Figure 5:**
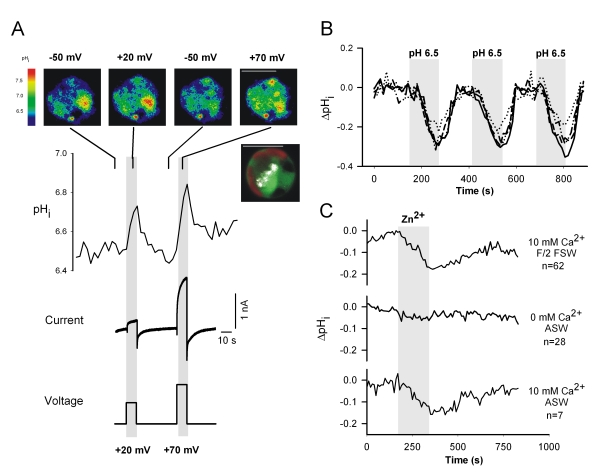
H^+^ conductance-mediated regulation of pH_i_. (A) Representative simultaneous whole cell patch clamp and pH_i_ imaging in *C. pelagicus* cells (300 µM BCECF free acid loaded via the patch pipette). Top panel displays false colour BCECF fluorescence ratio images of *C. pelagicus* during the voltage step protocol (+20, −50, and +70 mV). The inset (top right) indicates localization of BCECF (green), chlorophyll autofluorescence (red), and reflectance of an internal developing coccolith (white). Scale bar, 10 µm. Membrane depolarization to +20 mV or +70 mV from a holding potential of −50 mV caused an increase in pH_i_. The pipette solution contained (in mM) K-Glutamate 200, MgCl_2_ 5, EGTA 5 and Pipes 1.0 (P1b, Table S2), and the external solution was ASW containing (in mM) NaCl 450, KCl 8, MgCl_2_ 30, MgSO_4_ 16, CaCl_2_ 10, NaHCO_3_ 2, and HEPES 20 (E1, Table S2). (B) The effect of pH_o_ on pH_i_ in BCECF-loaded *C. pelagicus* cells. Changing pH_o_ from 8.0 to 6.5 induced a substantial and reproducible reduction in pH_i_. Traces from 4 individual cells are superimposed. (C) The effect of Zn^2+^ on pH_i_ in calcifying *C. pelagicus* cells. Cells loaded with BCECF-AM were perfused with either f/2 FSW media pH 8.0 (*n* = 62) or artificial seawater (ASW) pH 8.0, containing 0 mM or 10 mM Ca^2+^ (*n* = 28 and 7, respectively). 30 µM free Zn^2+^ was perfused for 2.5 min (grey box); averaged traces are shown.

As the biophysical characteristics of the outward conductance in *C. pelagicus* and H_v_1 channel homologues are consistent with those described for animal H_v_1 channels [17],[19]–[21], we hypothesized a role in rapid H^+^ efflux during pH homeostasis. Using simultaneous patch clamp and pH imaging, we verified that direct activation of the *C. pelagicus* H^+^ current induced significant changes in cytoplasmic pH (Figure 5A). The application of a 10 s depolarization more positive of E_H_^+^ activated the H^+^ current and induced significant reversible cytoplasmic alkalinisation (Figure 5A). The mean increases in pH_i_ following voltage steps from −50 mV to +20 or +70 mV were 0.22 (±0.04 SE) and 0.36 (±0.04 SE, *n* = 7) pH units, respectively. These results are consistent with H^+^ efflux through a voltage-sensitive conductance as described in animal systems (e.g., [21]). Following the depolarising pulses, pH_i_ recovered approximately exponentially over 30–60 s, which is faster but not substantially different from recovery times reported in animal cells [24]. The faster pH recovery reported here may reflect a number of factors, including the rate of diffusion of buffer from the patch electrode and a significantly lower relative cytoplasmic volume of coccolithophore cells which contain large chloroplasts, vacuoles, and coccolith vacuole. Hyperpolarizing pulses that activate a large inward Cl^-^ current [18] caused no significant pH_i_ change (Figure S5). These observations bear key similarities to results from a number of animal cell types in which H^+^ currents are known to mediate pH homeostasis and charge balance [17],[25],[26]. They provide strong evidence that the *C. pelagicus* outward H^+^ current can specifically and significantly contribute to regulation of cytoplasmic pH.

In order to understand how the outward H^+^ current operates to regulate cytoplasmic pH, we determined the resting membrane potential (V_m_) in *C. pelagicus*. Using sharp microelectrodes, V_m_ of intact *C. pelagicus* cells was measured at −45.7 mV (±4.8 SE, *n* = 10) in ASW at pH_o_ 8.0. At pH_o_ 6.5 V_m_ depolarised to −29.0 mV (±3.1 SE, *n* = 10). The measured V_m_ at pH_o_ 8.0 is very close to E_H_^+^ (−48 mV, assuming a resting pH_i_ of 7.2 typical of eukaryote cells), suggesting that the H^+^ conductance will be close to its activation potential under normal conditions.

In a model whereby channel-mediated H^+^ efflux regulates pH_i_, treatments that inhibit the H^+^ current may cause cytosolic acidification in calcifying cells. Accordingly, the vast majority of *C. pelagicus* cells (85%, *n* = 44, 7 independent experiments) showed a strong dependence of pH_i_ upon pH_o_, exhibiting reversible acidification when external pH was rapidly shifted from pH 8.0 to pH 6.5 (Figure 5B). This is consistent with the sensitivity of the H^+^ conductance to the H^+^ electrochemical gradient across the plasma membrane. The direct effect of pH_o_ on pH_i_ also implies that *C. pelagicus* is not able to maintain intracellular pH in the face of transient shifts in pH_o_, which is consistent with evolution in the open ocean where pH is relatively stable.

To address the requirement for the H^+^ conductance during calcification, we examined the effect of Zn^2+^ on pH_i_ in actively calcifying cells. Treatment with 30 µM free Zn^2+^ for 2.5 min caused an immediate decrease in cytosolic pH in calcifying cells (mean ΔpH  =  −0.13±0.02 SE, *n* = 62, Figure 5C). Conversely, in cells where calcification was inhibited by incubation in Ca^2+^-free artificial seawater [15] Zn^2+^ did not induce a large decrease in pH_i_ (mean ΔpH  =  −0.02±0.02 SE, *n* = 29, Figure 5C). The inward Cl^−^ rectifier is also sensitive to Zn^2+^ [18] but is unlikely to influence the observed acidification as inhibition of this conductance would act to depolarise V_m_ resulting in cytoplasmic alkalinisation. We conclude that the plasma membrane H^+^ conductance plays an important role in release of H^+^ from the cytosol in actively calcifying cells.

**Figure 6:**
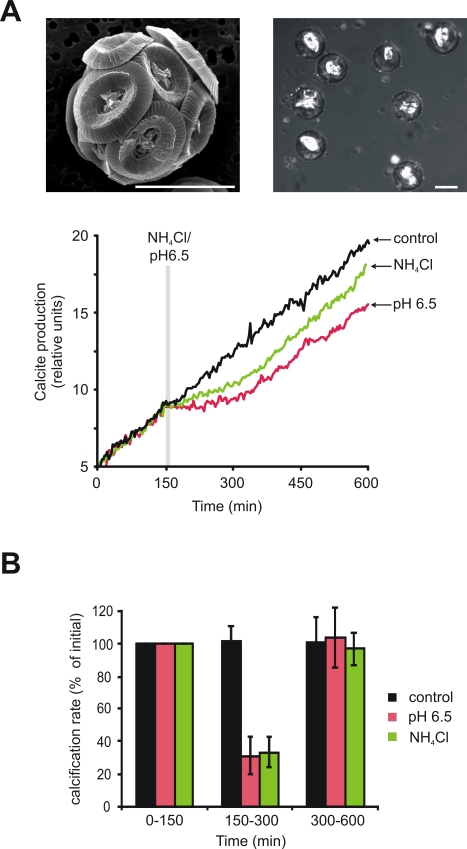
Calcification and pH_i_ regulation in *C. pelagicus.* (A) Calcification rate following manipulation of pH_i_. Coccolith production by decalcified *C. pelagicus* cells in f/2 FSW media (pH 8.2, 15 °C) was monitored by cross-polarised light microscopy (upper right and Figure S6). Upper left shows a scanning electron micrograph of a *C. pelagicus* cell with an intact coccosphere for reference. Scale bars, 10 µm. pH_i_ was manipulated by perfusion with f/2 media at pH 8.2 (control), at pH 6.5, or at pH 8.2 + 10 mM NH_4_Cl for 10 min (lower panel). Note the appreciable lag in restoration of the initial calcification rate following acidification of the cytosol. Traces are normalized to initial rate (0–150 min). (B) Mean calcification rate (±SE) was calculated by linear regression for each period (0–150, 150–300, and 300–600 min) and is expressed as a percentage of the initial rate (*n* = 4 except for pH 6.5, where *n* = 3).

**Figure 7:**
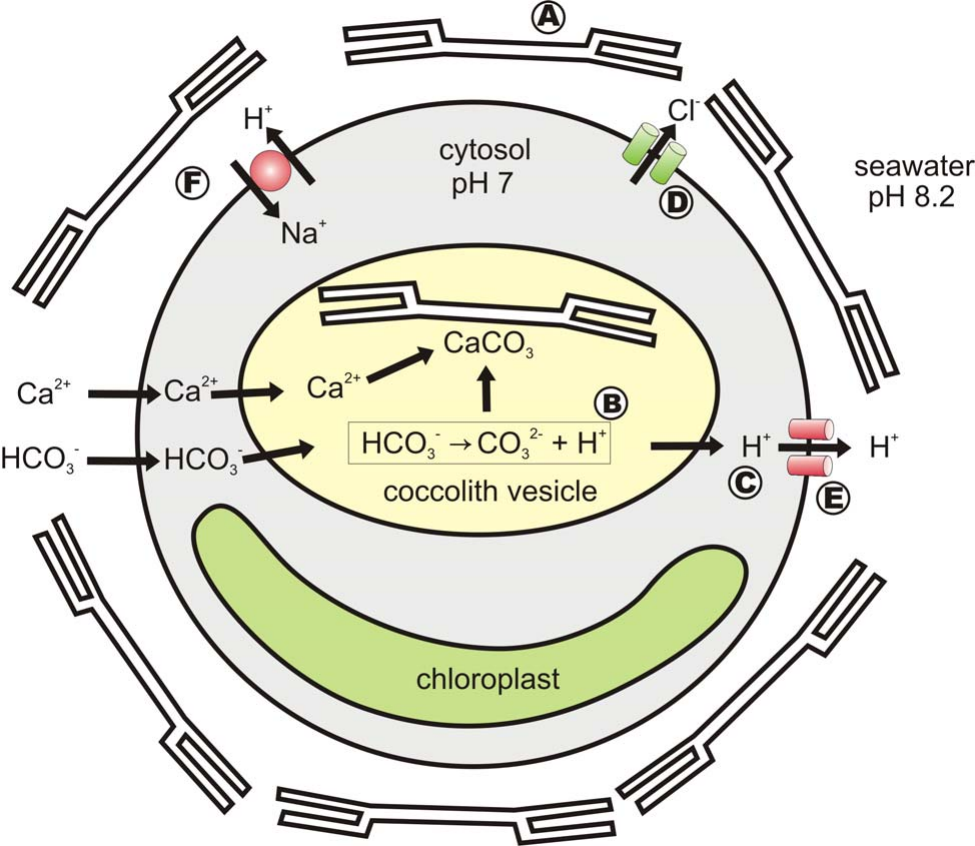
Model of the major ion fluxes associated with calcification and pH homeostasis in coccolithophores. The scheme illustrates the requirement for efficient H^+^ efflux pathways in coccolithophores as a result of intracellular calcification. Mature coccoliths are arranged on the extracellular surface, surrounding the cell to form a coccosphere (A). However, coccolith formation occurs within the intracellular Golgi-derived coccolith vacuole. Calcium carbonate (CaCO_3_) precipitation requires the production of carbonate (CO_3_^2−^) from bicarbonate (HCO_3_^−^) and results in the net production of H^+^ (B). H^+^ must be rapidly removed from the coccolith vacuole in order to maintain a suitable pH for CaCO_3_ precipitation. Once in the cytosol (C), some H^+^ may potentially be utilised by photosynthesis in the production of CO_2_ from HCO_3_^−^, however H^+^ efflux provides an efficient mechanism to prevent cytosolic acidosis during fluctuations in photosynthetic rate. At normal seawater pH 8.2, pH_i_ of 7.2, and V_m_ ∼−46 mV maintained by a Cl^–^ inward rectifier (D) [18], a drop in pH_i_ alone or in combination with membrane depolarisation would result in a net outward proton motive force across the plasma membrane. This enables passive H^+^ efflux via a plasma membrane localised H^+^ channel (E), providing a rapid mechanism for maintaining constant intracellular pH. Other membrane transporters (yet to be characterised) are likely involved in longer term maintenance of cytoplasmic pH (F). Nevertheless, the pH- and voltage-sensitive gating mechanism of the H^+^ channel coupled to its high transport capacity suggests it plays a major role in the modulation of intracellular pH in coccolithophores. Patch clamp studies indicate that Cl^–^ and H^+^ are the dominant transmembrane conductances in coccolithophores.

To address further the role of pH_i_ homeostasis during calcification, we manipulated pH_i_ whilst measuring calcification rates with a non-invasive in vivo method (Figure 6A, Figure S6). A reversible decrease in pH_i_ induced by a brief reduction in extracellular pH_o_ (10 min at pH_o_ 6.5, Figure S7) caused a 69.0% ± 11.4% inhibition of calcification rate (Figure 6A,B). As changes in pH_o_ also affect extracellular C_i_ speciation, we used a pulse of NH_4_Cl (10 mM, 10 min, Figure S7) to induce intracellular acidification while maintaining constant pH_o_ [27]. This resulted in a 67.0% ± 8.7% inhibition of calcification (Figure 6B). Inhibition of calcification continued for up to 2 h post-treatment, suggesting that down-regulation of the calcification machinery operates in response to disruption of pH_i_ (Figure 6B). Whilst indirect effects of pH_o_ and NH_4_Cl treatments may contribute to the inhibition of calcification, the similar effects of different treatments imply a direct relationship between pH_i_ homeostasis and calcification. The sensitivity of calcification to fluctuations in pH_i_ highlights the requirement for efficient regulation of pH_i_, which is in turn dependent on the voltage-gated H^+^ conductance, and strongly supports a role for the coccolithophore H_v_ channels as a key component in the calcification process (Figure 7).

## Discussion

In combination with the emerging genomic information, our data provide clear evidence for physiological features that are novel for photosynthetic eukaryotes. *C. pelagicus* expresses a homologue of animal voltage-gated H^+^ channels and exhibits an H^+^-selective conductance that is activated by depolarization and dependent upon the H^+^ electrochemical gradient. As with metazoan H^+^ channels [17],[28], the properties of the *C. pelagicus* H^+^ conductance appear ideally suited to mediating rapid H^+^ efflux during metabolic acidosis [28]. Our data also support a functional link between the coccolithophore H^+^ conductance and sustained intracellular calcification via regulation of pH_i_. Our electrophysiological and molecular analyses lead us to propose the following model (Figure 7). Calcification in coccolithophores is likely to generate H^+^ at a relatively constant rate, whereas the rates of H^+^ consumption by metabolic processes (e.g., photosynthesis) are likely to fluctuate rapidly (e.g., with changes in light intensity). The net H^+^ load resulting from calcification will therefore vary constantly. In calcifying coccolithophores, two dominant conductances are expressed in the plasma membrane: an inward Cl^−^ rectifier activated by hyperpolarisation and dependent upon E_Cl_ [18] and an outward H^+^ conductance activated by depolarisation and dependent upon E_H_^+^ (this study). Excursions of V_m_ more negative of resting V_m_ will have no effect on pH_i_ because only the inward Cl^−^ current is activated (as evidenced in Figure S5) and those positive of E_H_^+^ will induce outward H^+^ flow and changes in pH_i_ (see Figure 5). Our calculations suggest that the H^+^ conductance will be very close to its activation potential under normal conditions (i.e., at pH_o_ 8.0 and pH_i_ 7.2, V_m_ is −46 mV and E_H_^+^ is −48 mV). Cytoplasmic acidification would also activate H^+^ efflux by shifting E_H_^+^ more negative (i.e., independent of any change in V_m_). Therefore a calcifying cell in which H^+^ production is not balanced by metabolic H^+^ consumption will generate an acid load on the cytosol which will trigger activation of the H^+^ outward current. Combined depolarisation of V_m_ and acidification would act synergistically to induce potentially rapid H^+^ efflux and subsequent membrane hyperpolarization. This efflux may be sustained by activation of the sensitive inward Cl^−^ current (Cl^−^ efflux) at more negative V_m_ [18], which will balance the charge and facilitate maintenance of H^+^ removal during calcification. Thus, we propose that outward rectifying H_v_1 channels and inward rectifying Cl^−^ channels work together to sustain H^+^ efflux.

H_v_1 homologues are not universally present in marine algae, being absent from the genomes of prasinophytes (both *Ostreococcus* and *Micromonas*) and the brown macroalga, *Ectocarpus siliculosus*, suggesting that these channels play specialised cellular roles in coccolithophores and diatoms. Interestingly, a protein exhibiting weak similarity to EhH_v_1 is also present in the genomes of the moss *Physcomitrella patens* and other land plants, although in these predicted proteins a conserved arginine in S4 (corresponding to human R205) is replaced by a threonine residue. Moreover, a plasma membrane voltage-gated H^+^ conductance has not been described in land plants, indicating that such homologues play distinctly different functional roles in these organisms.

Comparative studies of H_v_1 proteins from such divergent eukaryote taxa have the potential to provide critical insight into the conserved features required for the novel mechanisms of H^+^ conductance within this group of ion channels (see Figure 3A–C) [17]. As H_v_1 proteins lack a classic pore, the mechanism of H^+^ permeation through H_v_1 proteins is not fully understood. Ionisable residues in the transmembrane domains may contribute to H^+^ conduction via a hydrogen bonded chain mechanism [17], although recent evidence from a combined mutagenesis and structural modelling approach suggests that H^+^ may be conducted via an internal water wire, rather than the ionisable side chains of transmembrane residues [29]. Analysis of the transmembrane domains of coccolithophore H_v_1 proteins indicates that the acidic residues are broadly conserved, along with the arginine residues associated with voltage gating in S4.

Physiological studies so far have not allowed a clear distinction between HCO_3_^−^ and direct CO_3_^2−^ uptake for calcification. However, this knowledge is critical for understanding the impacts of reduced ocean surface pH on calcification, because predicted changes in ocean chemistry will bring about significant reductions in ocean surface [CO_3_^2−^] [30]. The presence of an effective mechanism to dissipate excess H^+^ is consistent with HCO_3_^−^ as the primary seawater C_i_ substrate for calcification and suggests there is no obligate requirement for H^+^ derived from calcification to be utilized for intracellular CO_2_ generation.

The effects of elevated CO_2_/decreased seawater pH on coccolithophores vary according to species, strain, and experimental conditions [3]–[5],[31]. Understandably, much attention has been paid to the effects of a decrease in calcite saturation on the dissolution of extracellular coccoliths. However, our studies identify a mechanism through which predicted ocean pH scenarios [32],[33] may also have a direct impact on the intracellular production of coccoliths [4],[5],[34] via the pH-dependent properties of the plasma membrane H^+^ channel. An understanding of the combined effects of ocean acidification on both calcite saturation and intracellular pH homeostasis is likely to be critical for unravelling the factors underlying the variation seen in laboratory studies of coccolithophore responses to ocean acidification. Based upon molecular phylogenetic evidence, coccolithophores evolved ∼250 MYA, with the earliest heterococcolith fossils dating between 204 and 217 MYA [35],[36]. Over this time period the oceans have remained supersaturated with regard to calcite, although surface ocean pH likely varied within the range of pH 7.6–8.2 [37], suggesting ancestral coccolithophores have previously experienced and survived in significantly lower ocean pH. The gating dependence of the H^+^ current on membrane ΔpH implies that its activity may be negatively impacted by reduced seawater pH. The expression and biophysical properties of the coccolithophore H^+^ conductance are likely to be important factors in determining how coccolithophores may themselves respond to predicted future changes in ocean pH. The sensitivity of calcification to transient changes in cytoplasmic pH is also evident from our results. This suggests an additional level of control of calcification whereby the calcification machinery may shut down under conditions where rapid control of cytoplasmic pH is compromised, relieving the cell of additional H^+^ load.

Whilst the H^+^ conductance is strictly dependent on the transmembrane H^+^ gradient, the gating properties of the H^+^ conductance and the magnitude of H^+^ flux described here are also under tight control of membrane potential. This may impart a degree of tolerance and physiological plasticity to the calcification process since effects of decreased pH_o_ on H^+^ efflux may be countered by slight adjustments of membrane voltage to maintain H^+^ efflux.

## Materials and Methods

### Cell Culturing

Batch cultures of the unicellular haptophyte alga *Coccolithus pelagicus* ssp *braarudii* (PLY 182G, from the Plymouth Culture Collection) were grown in either filtered seawater (FSW) or artificial seawater (ASW: 450 mM NaCl, 30 mM MgCl_2_, 16 mM MgSO_4_, 8 mM KCl, 10 mM CaCl_2_, and 2 mM NaHCO_3_), supplemented with nutrients, trace metals, and vitamins [18]. Cultures were maintained at 15°C under 100 µmol m^2^ s^−1^ light on a 16∶8 h light:dark cycle. Under these growth conditions the cell diameter was between 10 and 15 µm. Before electrophysiological and optical recordings, cells were decalcified with brief EGTA treatment followed by trituration as previously described [18], which removed the external calcite coccosphere and body scales. The resultant protoplasts (7–12 µm in diameter, Figure S1) allowed patch clamp recording and improved the optical properties of the cells for imaging. This brief treatment did not affect subsequent cellular calcification and growth rates [10],[18].

### *C. pelagicus* Patch-Clamp Recording and Analysis

Whole cell patch clamp recordings were conducted at 20°C as previously described [18]. The recording chamber volume was 1.5 cm^3^, and solutions exchanged using gravity-fed input and suction output at a rate of 5 cm^3^ min^−1^. All pipette solutions contained EGTA, HEPES, or PIPES buffer and sorbitol to balance the osmolarity to between 1,000 and 1,200 mOsmol kg^−1^. Specific ionic compositions of bath and pipette solutions were chosen to give optimal buffering or pH responses and are given in Table S2 and in the figure legends. Liquid junction potentials were calculated using the junction potential tool in Clampex (Molecular Devices, Sunnyvale, CA) and corrected off-line. Whole cell capacitance and seal resistance (leak) were periodically monitored during experiments by applying a <5 mV test pulse. Currents were linear leak subtracted in Clampfit (Molecular Devices, Sunnyvale, CA) using the pre-test seal resistance. Current voltage relations were determined on leak subtracted families by measuring the maximum steady state amplitude (averaging between 10 and 50 ms of the current trace). Reversal potentials were determined by manually measuring the peak tail currents of leak subtracted families of traces and calculating a linear regression versus test voltage. Series resistance was monitored throughout the experiments and whole cell currents were analysed only from recordings in which series resistance varied by less than 15%.

### Intracellular pH Measurement

Decalcified *C. pelagicus* cells were either loaded by incubation of between 20 and 40 min at 20°C with 5 µM BCECF-AM (Invitrogen, Paisley, UK) or by inclusion of 300 µM BCECF free acid in the patch clamp pipette (solution P1b, Table S2). Changes in intracellular pH were monitored by the ratio of the fluorescence emission at 525±25 nm when excited sequentially with 488 and 458 nm (LSM 510 confocal microscope, Zeiss, Jena, Germany). For each excitation wavelength, the average fluorescence intensity was determined for a region of interest encompassing the whole cell and used to calculate the ratio. Background fluorescence was minimal and was not subtracted. In patch clamp experiments, pH was calibrated using mean steady state fluorescence ratio (F_488_/F_458_) at pH_i_ 7.5 and pH_i_ 6.5 (*n* = 10 and *n* = 5, respectively). We were unable to achieve a satisfactory calibration for ester-loaded cells using the nigericin technique as BCECF fluorescence was not stable in *C. pelagicus* in the presence of this protonophore. Absolute pH values are therefore not given for ester-loaded cells; rather ΔpH values are given, calculated from the calibration performed in patch-loaded cells with pH_i_ set by the pipette solution. The magnitude of ΔpH calculated in this manner closely matched ΔpH calculated from an in vitro calibration curve using 25 µM BCECF free acid in “cytosolic” buffer solutions (15 mM HEPES, 15 mM MES, 1 mM MgCl_2_, 130 mM KCl, pH 6.5–8.5). Location of intracellular coccoliths was determined by imaging in reflectance mode (633 nm excitation) of the confocal microscope. Chloroplasts were visualised with 488 nm excitation and emission >600 nm.

### Cloning and Heterologous Expression

*EhHVCN1* (JGI protein ID: 631975) was identified by sequence similarity searches using animal H_v_1 to query the *E. huxleyi* genome (Joint Genome Institute, http://genome.jgi-psf.org/Emihu1/Emihu1.home.html). *CpHVCN1* was identified in a collection of ESTs generated from *C. pelagicus spp braaudii* strain LK1 by Peter von Dassow and co-workers at the Station Biologique de Roscoff, France (Genbank accession no. HM560965). To confirm expression and the coding sequence of these genes, 1.1 kb cDNAs corresponding to the open reading frame of *EhHVCN1* or *CpHVCN1* were amplified by reverse transcription-polymerase chain reaction from *E. huxleyi* strain CCMP1516 or *C. pelagicus* strain PLY 182 g using the primers EhHVCN1_F3 TCATCCCTCTCTTTGCGATG with EhHVCN1_R3 GGTCTTTGGAAACGGTTAGC and CpHVCN1_F7 GCAAATATTTTAGAAGGATGAGG with CpHVCN1_R4 GAGATTTGAACACGCGAAT. Due to the high GC content (*E. huxleyi*) and unusual codon usage (both species), we synthesised codon-optimised versions of the transcripts for characterisation in mammalian expression systems (GenScript, Piscataway, NJ). These inserts were subcloned into pcDNA3.1 via *HindIII* and *XbaI*. For electrophysiology, HEK293 cells were transiently co-transfected with 1.0 µg of pcDNA3.1 *HVCN1* plus 0.4 µg of pCDNA3.1-eGFP using Lipofectamine LTX (Invitrogen). To confirm *EhHVCN1* and *CpHVCN1* were expressed in HEK293 cells, these genes were subcloned into a pcDNA3.1-eGFP construct via *HindIII* and *BamHI* sites to generate green fluorescent protein (GFP) fusions (*EhHVCN1-GFP*, *CpHVCN1-GFP*). HEK293 cells transfected with the GFP-fusions demonstrated localisation to both the plasma membrane and endomembranes for both proteins and exhibited very similar currents to the non-fusion proteins during patch-clamp recordings. For all further characterisations with the exception of the site-directed mutagenesis, *EhHVCN1* or *CpHVCN1* alone were used 24–48 h after transfection. Site-directed mutagenesis of histidine residues in *EhHVCN1-GFP* was performed using a QuikChange II Site-Directed Mutagenesis kit (Stratagene, La Jolla, CA). All mutagenesis products were confirmed by DNA sequencing.

### Protein Alignments and Phylogenetic Analysis

Amino acid sequences of proteins were aligned using ClustalW. Accession numbers and protein length for H_v_1 proteins are as follows: Homo sapiens (NP_001035196, 273aa), *Mus musculus* (NP_083028, 269aa), *Gallus gallus* (NP_001025834, 235aa), *Danio rerio* (NP_001002346, 235aa), *Xenopus laevis* (NP_001088875, 230aa), *Ciona intestinalis* (NP_001071937, 342 aa), *Emiliania huxleyi* (JGI v1.0 prot ID: 631975, 339aa), *Coccolithus pelagicus* (HM560965, 325aa), *Phaeodactylum tricornutum* (XP_002180795, 338aa), *Thalassiosira pseudonana* (XP_002293360.1, 293aa), *Polysphondylium pallidum* (EFA75681.1, 280aa), and *Physcomitrella patens* (XP_001767834.1, 198aa). Accession numbers for additional proteins used are as follows: *Drosophila melanogaster - Shaker* (NP_728122.1), *Homo sapiens - K_v_2.2* (AF450111), *Arabidopsis thaliana - KAT1* (NP_199436.1), *Aeropyrum pernix K1* - *KvAP* (NP_147625.1), and *Bacillus halodurans C-125 - NaChBac* (NP_242367.1). For the phylogenetic analysis, an alignment was constructed based on the conserved residues surrounding the four transmembrane domains. Sequences were aligned using MUSCLE and then manually corrected to ensure only unambiguous residues were compared. Maximum likelihood phylogenetic analysis was performed using PhyML within the Bosque software package [38], based on the JTT substitution matrix [39]. One hundred bootstrap replicates were performed.

### HEK293 Cell Electrophysiology

HEK293 cells (Health Protection Agency Culture Collection, Salisbury, UK) were maintained in Dulbecco's Modified Eagle Medium at 37°C in 5% CO_2_. Whole cell patch clamp recordings were performed at 20°C. HEK293 cells transfected with pCDNA3.1-eGFP alone were used as a control. The intracellular and extracellular solutions were based on those used by Sasaki et al. [20] (see legends for Figures 4, S4 and Table S2).

### In Vivo Calcification Monitoring

Calcification rate in *C. pelagicus* cells was quantified in vivo by monitoring the degree of birefringence of calcite using cross-polarized light microscopy. Cells were initially perfused with Ca^2+^-free ASW supplemented with 20 mM EGTA until decalcified, followed by perfusion with f/2 FSW and recovery for 2–3 h prior to imaging. Image capture was performed using a Nikon Diaphot microscope equipped with an Orca-100 cooled CCD camera (Hamamatsu Photonics, Shizuoka, Japan). All recordings were performed at 15°C, light intensity 110 µmol m^−2^ s^−1^. Time-lapse images were captured at a frame rate of 20 images h^−1^. The change in grey scale image intensity, which is proportional to production of birefringent calcite, was determined using LSM Image Examiner software (Zeiss).

## Supporting Information

Figure S1 Light micrographs of calcified and decalcified *C. pelagicus* used for electrophysiology. Top panel are calcified cells. Lower panel cells have been decalcified in buffered EGTA artificial seawater. Note in the lower panel a patch clamp electrode attached to the decalcified cell containing a mature intracellular coccolith. Scale bar, 10 µm.
(TIF)

Figure S2 The effect of pH_i_ on *C. pelagicus* H^+^ currents. Whole cell currents from *C. pelagicus* cells in response to incremental 600 ms depolarisations from −80 to +60 mV at different patch pipette pH values. (A) Families of whole cell currents recorded in two different cells in response to depolarising steps from a holding potential of −60 mV to +100 mV (shown for 20 mV increments). The pH of the external ASW solution was 8.0 (E1, Table S2). Patch pipette solutions contained 150 mM HEPES and the pH of the internal solutions was 7.5 (top traces) and 6.5 (bottom traces). (B) Mean (±SE) current-voltage relationships (10 mV increments) for the current measured at the end of the 600 ms pulse with internal pH of 7.5 (filled circle, *n* = 12) and 6.5 (filled triangle, *n* = 15).
(TIF)

Figure S3 H^+^ channel block by trivalent cations. (A) Currents recorded in response to a voltage step to +85 mV from a holding potential of −75 mV illustrating the complete block of the voltage activated outward current (control) after the addition of Gd^3+^ (100 µM). Pipette solution was 200 mM K-glutamate pH 7.5 (solution P1a), and bath solution was ASW pH 8.0 (solution E1) as detailed in Figure 1A. (B) Average current voltage curves for voltage activated outward current in ASW (filled circles) and after the application of 100 µM Gd^3+^ (open circles), *n* = 3.
(TIF)

Figure S4 Histidine residues required for Zn^2+^ inhibition of EhH_v_1. Predicted external histidine residues that are conserved across algal H_v_1 proteins were mutated in EhH_v_1-GFP to examine whether these residues played a role in the inhibition of the H^+^ conductance by Zn^2+^. *EhHVCN1-GFP* (control), *H197A*, and *H203A* were expressed in HEK293 cells and currents were recorded in response to a voltage step from −60 mV to 100 mV. 500 µM Zn^2+^ was added to the external solution. The pipette solution contained (in mM) NMDG 65, MgCl_2_ 3, EGTA 1, and HEPES 150 glucose 70, pH 7.0 (P4, Table S2), and the bath solution contained in (mM) NMDG 75, MgCl_2_ 3, CaCl_2_ 1.0, glucose 160, and HEPES 100, pH 7.8 (E4, Table S2).
(TIF)

Figure S5 Hyperpolarisation of the *C. pelagicus* plasma membrane does not induce an increase in pH_i_. Simultaneous patch clamp and pH imaging was performed in order to examine the effect of hyperpolarisation on pH_i_. Decalcified cells were loaded via the patch pipette with 300 µM BCECF free acid. A voltage step to −90 mV or −110 mV from a holding potential of −50 mV does not result in a change in pH_i_. Hyperpolarisation therefore activates a significant inward current (the Cl^−^ inward rectifier), but this current does not influence pH_i_. A representative of three replicate experiments is shown. Internal and external solutions are as used in Figure 5A (P1b, E1).
(TIF)

Figure S6 Determination of in vivo calcification rate by cross-polarized light microscopy. The figure shows the increase in cross-polarized light intensity monitored as decalcified cells produce coccoliths and the resultant calcite accumulates in the field of view. Stills from the time-lapse video illustrate the increase in grey-scale intensity during the 20 h incubation. Initial cross-polarised light intensity level at the start of the plot is due to the presence of internal coccoliths which are not removed by the decalcification protocol. The birefringence of calcite enables real time imaging of coccolith production. Birefringence in initial images is due to the presence of internal coccoliths which are not removed by the decalcification protocol. Time-lapse images were captured at a frame rate of 20 images h^−1^. Scale bar, 100 µm.
(TIF)

Figure S7 Manipulation of intracellular pH in *C. pelagicus*. In order to verify the effect of NH_4_Cl and pH_o_ treatments on pH_i_, cells were loaded with the pH responsive fluorescent dye BCECF and perfused with f/2 seawater media at pH 8.2. pH_i_ was manipulated by perfusion with either f/2 media at pH 6.5 (upper trace, representative of 10 experiments) or f/2 media containing 10 mM NH_4_Cl pH 8.2 (lower trace, representative of 15 experiments) for 10 min.
(TIF)

Table S1 Estimates of H^+^ production and potential acidosis during coccolithophore calcification.
(DOC)

Table S2 Composition of electrophysiology solutions (mM). NMDG, N-methyl-D-glutamine; E, external; P, pipette. In some experiments pH was adjusted to pH 6.5 for these solutions and HEPES was replaced with PIPES. For *C. pelagicus*, osmolarity was brought to between 1,000 and 1,200 mOsmol kg^−1^ by adding sorbitol. For HEK 293 cells, the osmolarity was adjusted 290–300 mOsmol kg^−1^ with glucose.
(DOC)
