# Supplementary figures and images for: A Voltage-Gated H+ Channel Underlying pH Homeostasis in Calcifying Coccolithophores
Source: PLoS Biol. 2011 Jun 21;9(6):e1001085. doi: 10.1371/journal.pbio.1001085 (PMC3119654; doi:10.1371/journal.pbio.1001085)

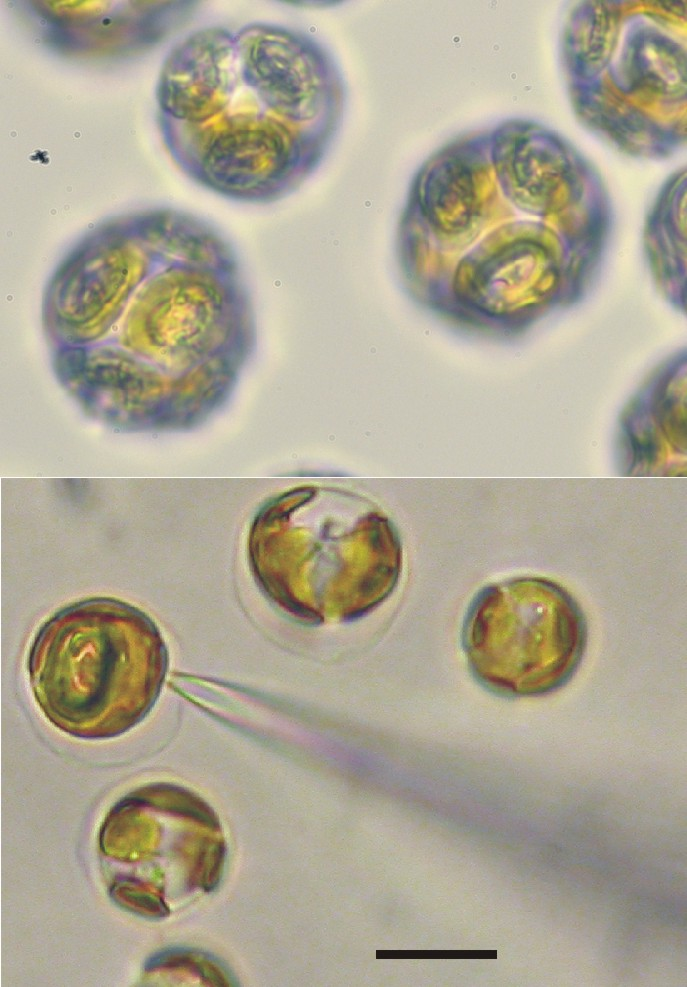

Supplement: Figure S1 — Light micrographs of calcified and decalcified C. pelagicus used for electrophysiology. Top panel are calcified cells. Lower panel cells have been decalcified in buffered EGTA artificial seawater. Note in the lower panel a patch clamp electrode attached to the decalcified cell containing a mature intracellular coccolith. Scale bar, 10 µm. (TIF) [file pbio.1001085.s001.tif]

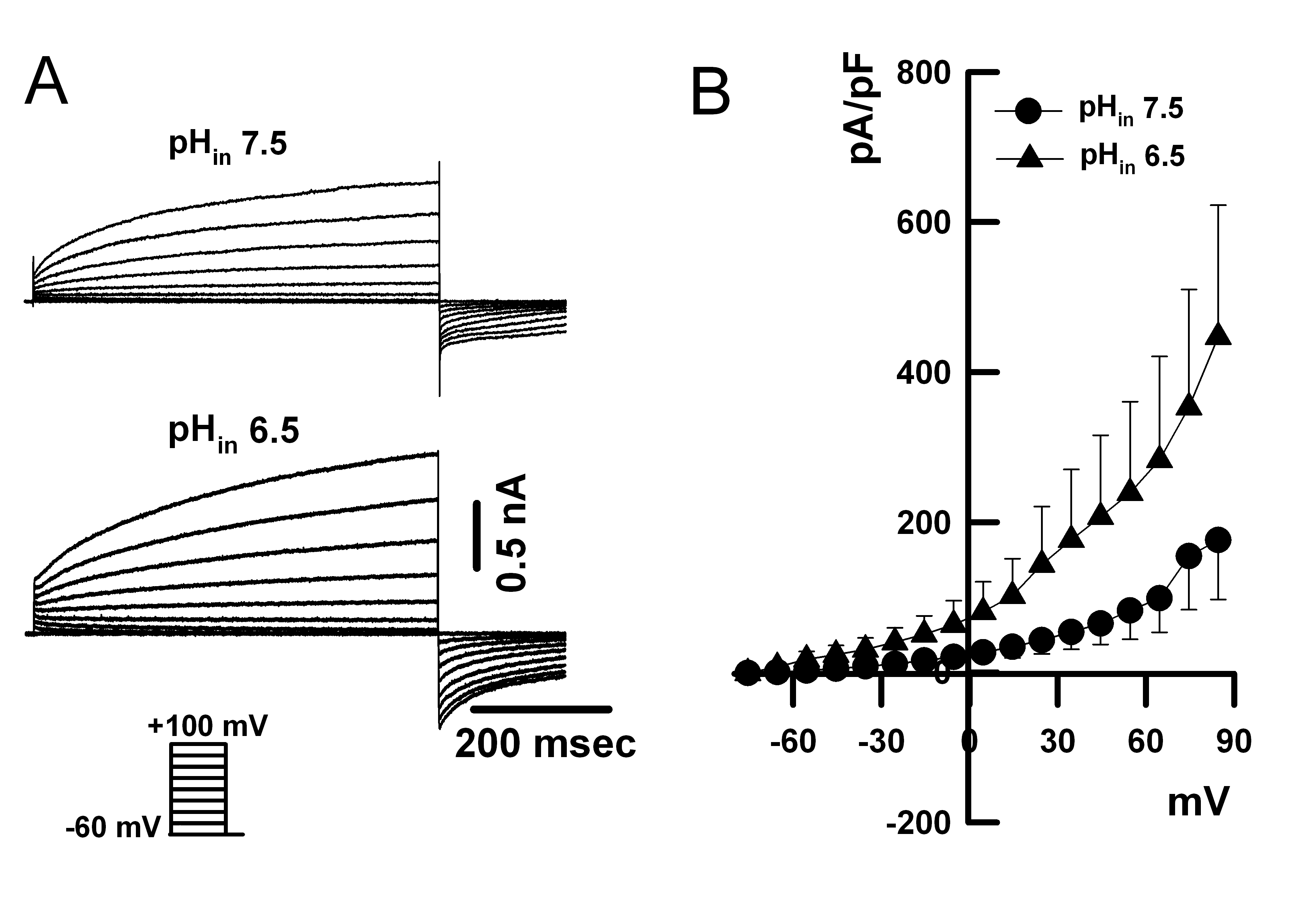

Supplement: Figure S2 — The effect of pHi on C. pelagicus H+ currents. Whole cell currents from C. pelagicus cells in response to incremental 600 ms depolarisations from −80 to +60 mV at different patch pipette pH values. (A) Families of whole cell currents recorded in two different cells in response to depolarising steps from a holding potential of −60 mV to +100 mV (shown for 20 mV increments). The pH of the external ASW solution was 8.0 (E1, Table S2). Patch pipette solutions contained 150 mM HEPES and the pH of the internal solutions was 7.5 (top traces) and 6.5 (bottom traces). (B) Mean (±SE) current-voltage relationships (10 mV increments) for the current measured at the end of the 600 ms pulse with internal pH of 7.5 (filled circle, n = 12) and 6.5 (filled triangle, n = 15). (TIF) [file pbio.1001085.s002.tif]

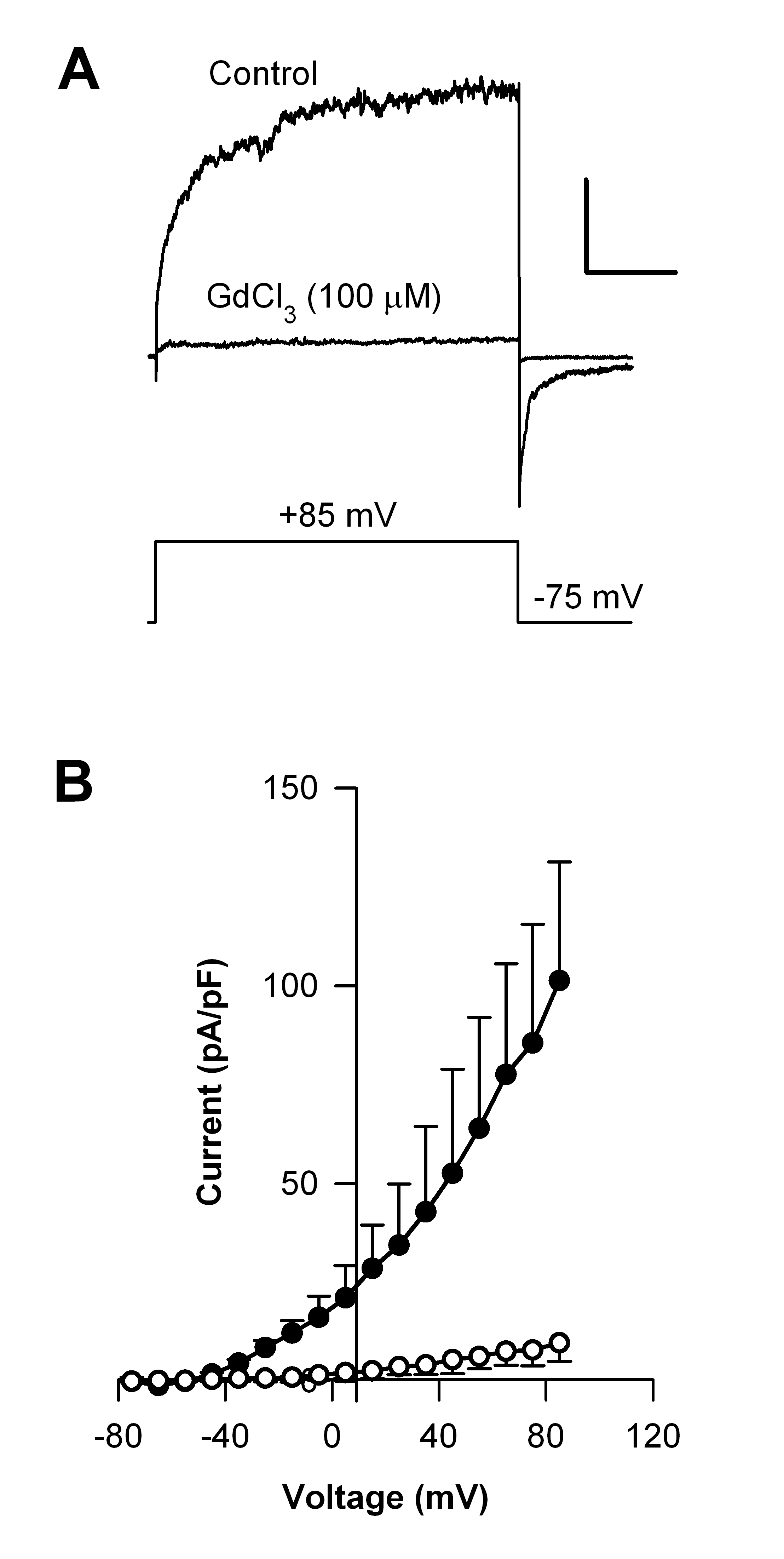

Supplement: Figure S3 — H+ channel block by trivalent cations. (A) Currents recorded in response to a voltage step to +85 mV from a holding potential of −75 mV illustrating the complete block of the voltage activated outward current (control) after the addition of Gd3+ (100 µM). Pipette solution was 200 mM K-glutamate pH 7.5 (solution P1a), and bath solution was ASW pH 8.0 (solution E1) as detailed in Figure 1A. (B) Average current voltage curves for voltage activated outward current in ASW (filled circles) and after the application of 100 µM Gd3+ (open circles), n = 3. (TIF) [file pbio.1001085.s003.tif]

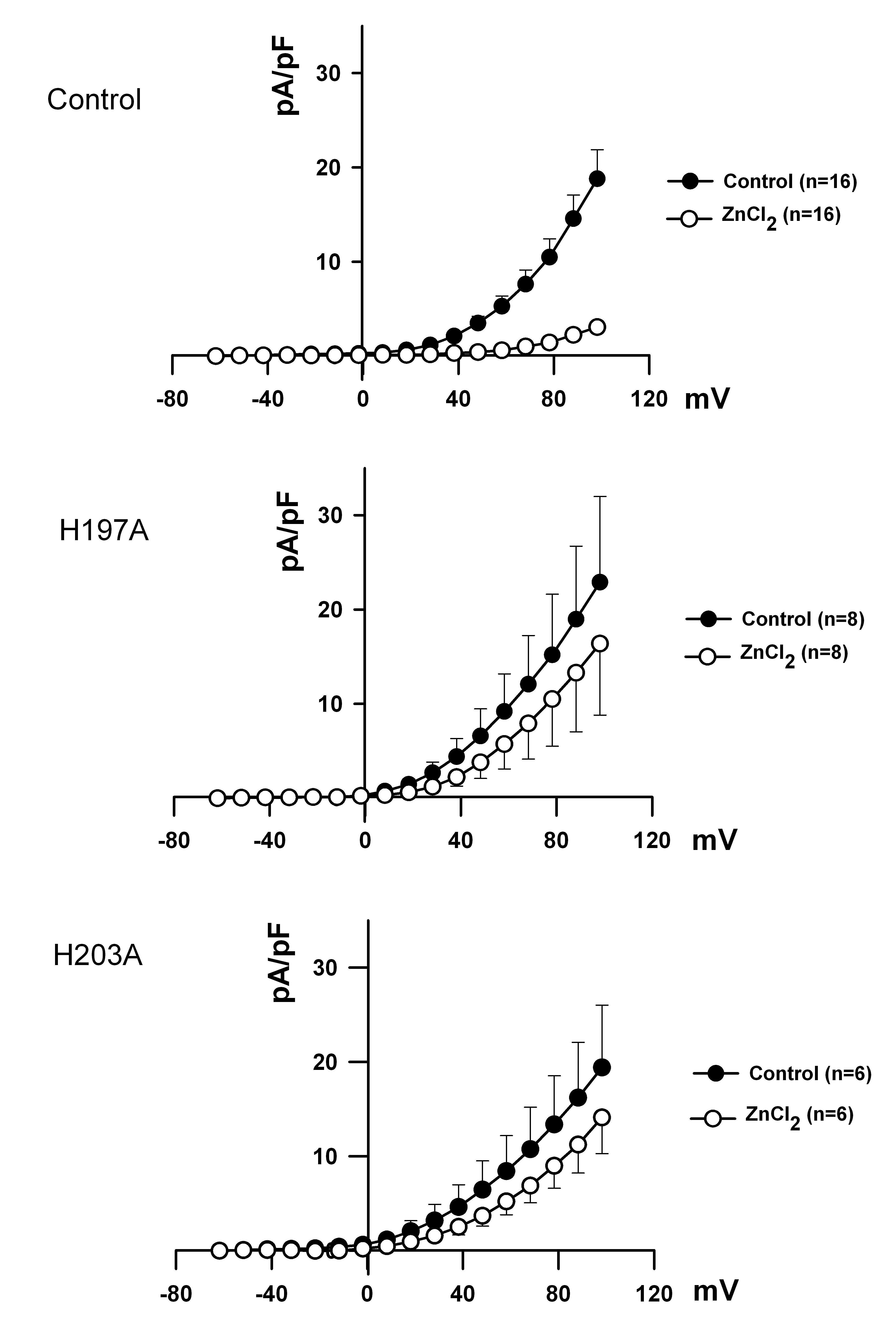

Supplement: Figure S4 — Histidine residues required for Zn2+ inhibition of EhHv1. Predicted external histidine residues that are conserved across algal Hv1 proteins were mutated in EhHv1-GFP to examine whether these residues played a role in the inhibition of the H+ conductance by Zn2+. EhHVCN1-GFP (control), H197A, and H203A were expressed in HEK293 cells and currents were recorded in response to a voltage step from −60 mV to 100 mV. 500 µM Zn2+ was added to the external solution. The pipette solution contained (in mM) NMDG 65, MgCl2 3, EGTA 1, and HEPES 150 glucose 70, pH 7.0 (P4, Table S2), and the bath solution contained in (mM) NMDG 75, MgCl2 3, CaCl2 1.0, glucose 160, and HEPES 100, pH 7.8 (E4, Table S2). (TIF) [file pbio.1001085.s004.tif]

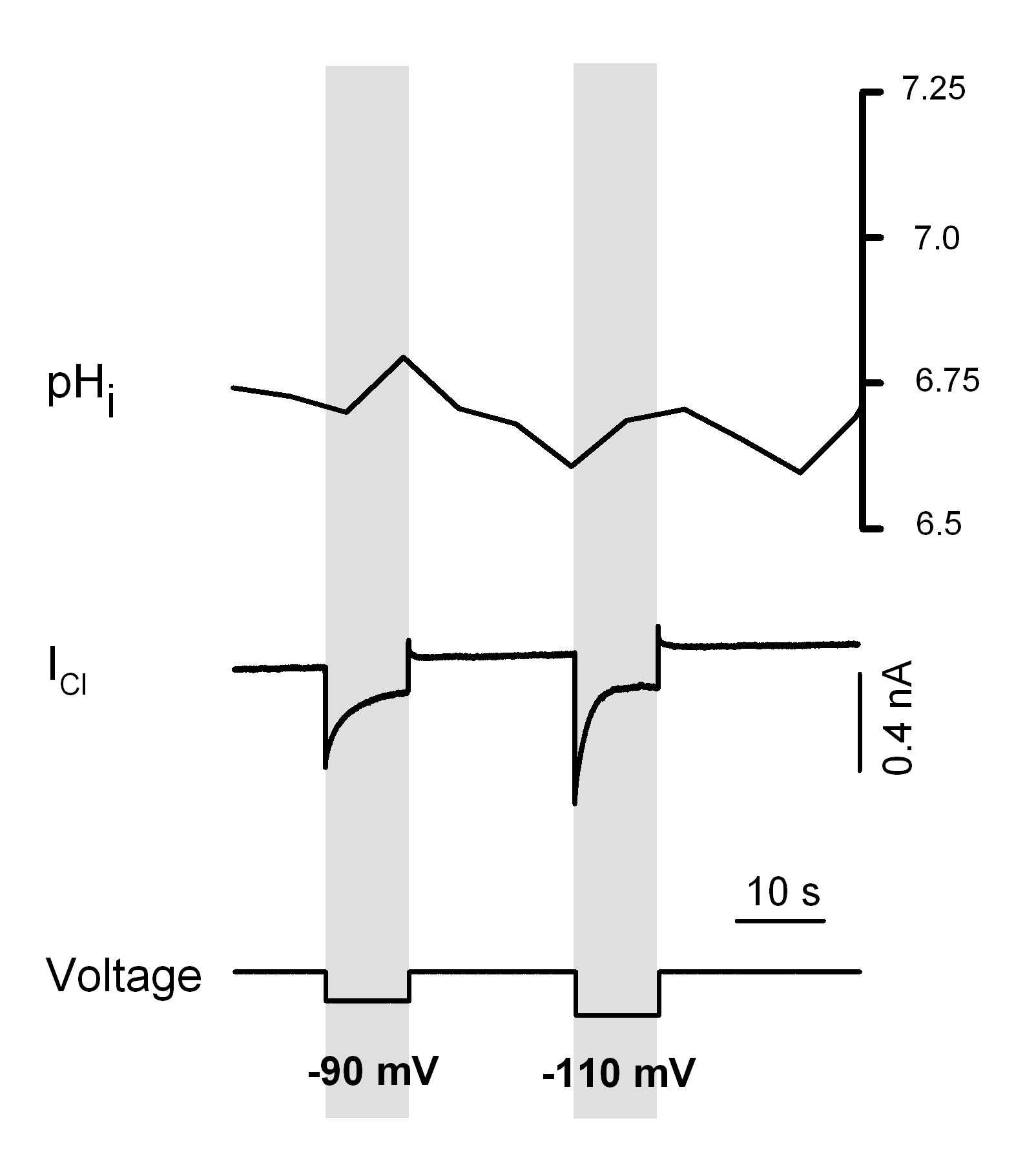

Supplement: Figure S5 — Hyperpolarisation of the C. pelagicus plasma membrane does not induce an increase in pHi. Simultaneous patch clamp and pH imaging was performed in order to examine the effect of hyperpolarisation on pHi. Decalcified cells were loaded via the patch pipette with 300 µM BCECF free acid. A voltage step to −90 mV or −110 mV from a holding potential of −50 mV does not result in a change in pHi. Hyperpolarisation therefore activates a significant inward current (the Cl− inward rectifier), but this current does not influence pHi. A representative of three replicate experiments is shown. Internal and external solutions are as used in Figure 5A (P1b, E1). (TIF) [file pbio.1001085.s005.tif]

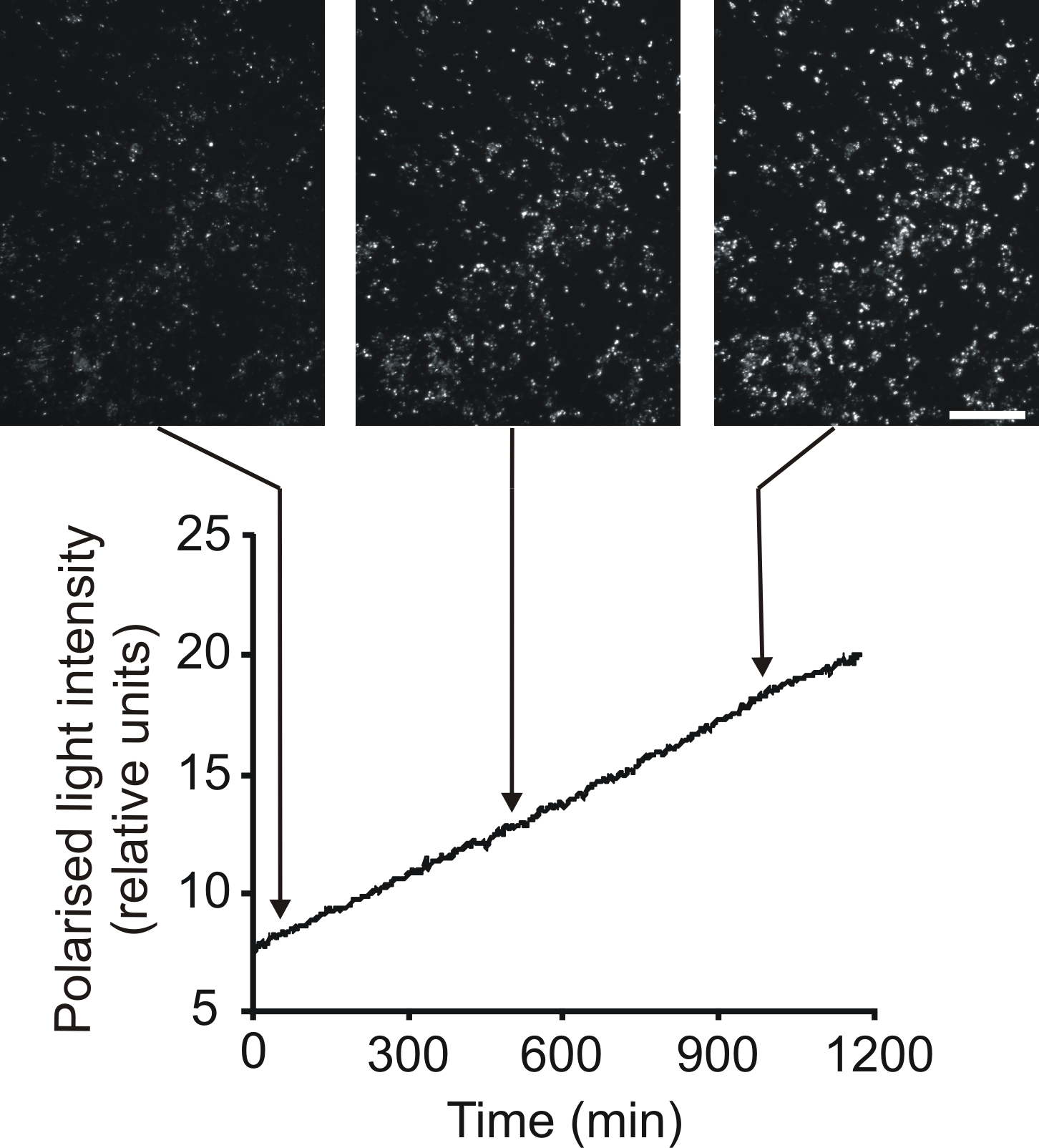

Supplement: Figure S6 — Determination of in vivo calcification rate by cross-polarized light microscopy. The figure shows the increase in cross-polarized light intensity monitored as decalcified cells produce coccoliths and the resultant calcite accumulates in the field of view. Stills from the time-lapse video illustrate the increase in grey-scale intensity during the 20 h incubation. Initial cross-polarised light intensity level at the start of the plot is due to the presence of internal coccoliths which are not removed by the decalcification protocol. The birefringence of calcite enables real time imaging of coccolith production. Birefringence in initial images is due to the presence of internal coccoliths which are not removed by the decalcification protocol. Time-lapse images were captured at a frame rate of 20 images h−1. Scale bar, 100 µm. (TIF) [file pbio.1001085.s006.tif]

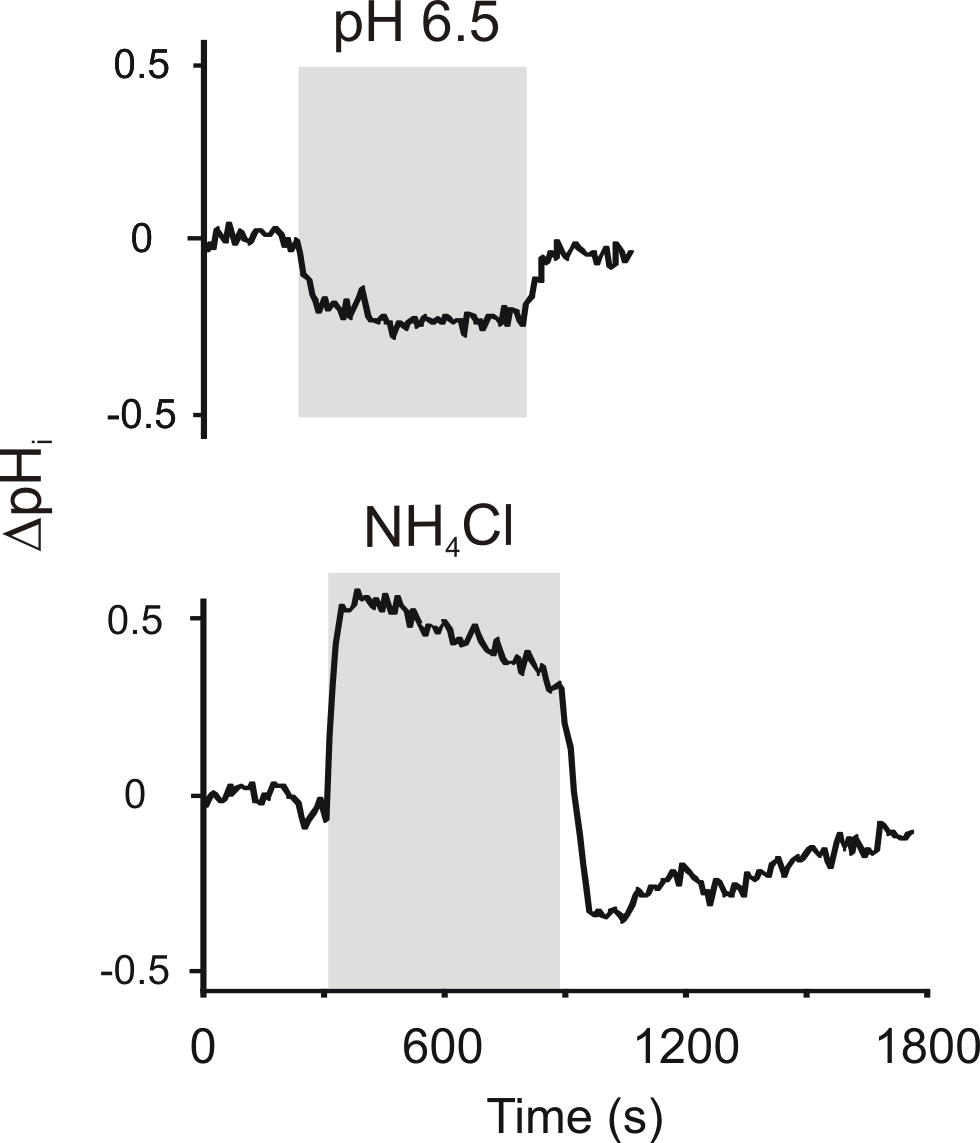

Supplement: Figure S7 — Manipulation of intracellular pH in C. pelagicus. In order to verify the effect of NH4Cl and pHo treatments on pHi, cells were loaded with the pH responsive fluorescent dye BCECF and perfused with f/2 seawater media at pH 8.2. pHi was manipulated by perfusion with either f/2 media at pH 6.5 (upper trace, representative of 10 experiments) or f/2 media containing 10 mM NH4Cl pH 8.2 (lower trace, representative of 15 experiments) for 10 min. (TIF) [file pbio.1001085.s007.tif]
