# Supplementary material for: A Voltage-Gated H+ Channel Underlying pH Homeostasis in Calcifying Coccolithophores
Source: PLoS Biol. 2011 Jun 21;9(6):e1001085. doi: 10.1371/journal.pbio.1001085 (PMC3119654; doi:10.1371/journal.pbio.1001085)
Supplement: Table S1 — Estimates of H+ production and potential acidosis during coccolithophore calcification. (DOC) [file pbio.1001085.s008.doc]

**Table S1. Estimates of H^+^ production and potential acidosis during coccolithophore calcification**

| **Species** | **Literature Source** | **Calcification Rate cell^-1^ s^-1^ (equivalent to H^+^ production)** | **Cell Volume^a^ (l)** | **Acidosis^b^ ΔpH min^-1^** | **H^+^ current^c^**  **pA cell^-1^** |
| --- | --- | --- | --- | --- | --- |
| *Emiliania huxleyi* | [1] | 3.38 x 10^-18^ mol Ca^2+^ | 3.35 x 10^-14^ | 0.30 | 0.33 |
|  | [2] | 4.86 x 10^-18^ mol calcite | 3.35 x 10^-14^ | 0.44 | 0.47 |
|  | [3] | 2.78 x 10^-18^ mol calcite | 3.35 x 10^-14^ | 0.25 | 0.27 |
|  | [4] | 5.09 x 10^-18^ mol C | 3.35 x 10^-14^ | 0.46 | 0.49 |
|  | [5] | 2.8 x 10^-18^ mol C | 3.35 x 10^-14^ | 0.25 | 0.27 |
|  | [6] | 4.63 x 10^-18^ mol C | 3.35 x 10^-14^ | 0.41 | 0.45 |
|  | [7] | 1.9 x 10^-18^ mol C | 3.35 x 10^-14^ | 0.17 | 0.18 |
| *Coccolithus. pelagicus* | [2] | 9.25 x 10^-17^ mol calcite | 9.05 x 10^-13^ | 0.31 | 8.92 |

^a^ Assuming 4 µm and 12 µm diameter for *E.huxleyi* and *C.pelagicus* respectively

^b^ Assuming cytoplasmic buffering capacity of 20 mM ΔpH [8] and in the absence of H^+^ removal and consumption

^c^ Steady state current (removal) required to balance H^+^ production in the absence of metabolic consumption

1. E. Paasche, S. Brubak, Enhanced Calcification in the Coccolithophorid *Emiliania huxleyi* (Haptophyceae) under Phosphorus Limitation. Phycologia **33**, 324 (1994).

2. H. M. Stoll, C. M. Klaas, I. Probert, J. Ruiz Encinar, J. I. Garcia Alonso, Calcification rate and temperature effects on Sr partitioning in coccoliths of multiple species of coccolithophorids in culture. Global and Planetary Change **34**, 153 (2002).

3. G. Langer et al., Species-specific responses of calcifying algae to changing seawater carbonate chemistry. Geochemistry Geophysics Geosystems **7**, (2006).

4. N. A. Nimer, M. J. Merrett, Calcification Rate in *Emiliania huxleyi* (Lohmann) in Response To Light Nitrate and Availability of Inorganic Carbon. New Phytologist **123**, 673 (1993).

5. M. N. Muller, A. N. Antia, J. LaRoche, Influence of cell cycle phase on calcification in the coccolithophore *Emiliania huxleyi*. Limnology and Oceanography **53**, 506 (2008).

6. W. M. Balch, J. Fritz, E. Fernandez, Decoupling of calcification and photosynthesis in the coccolithophore *Emiliania huxleyi* under steady-state light-limited growth. Marine Ecology-Progress Series **142**, 87 (1996).

7. L. Herfort, E. Loste, F. Meldrum, B. Thake, Structural and physiological effects of calcium and magnesium in *Emiliania huxleyi* (Lohmann) Hay and Mohler. Journal of Structural Biology **148**, 307 (2004).

8. A. Roos, W. F. Boron, Intracellular pH. Physiological Reviews **61**, 296 (1981).
