# Supplementary material for: A Voltage-Gated H+ Channel Underlying pH Homeostasis in Calcifying Coccolithophores
Source: PLoS Biol. 2011 Jun 21;9(6):e1001085. doi: 10.1371/journal.pbio.1001085 (PMC3119654; doi:10.1371/journal.pbio.1001085)
Supplement: Table S2 — Composition of electrophysiology solutions (mM). NMDG, N-methyl-D-glutamine; E, external; P, pipette. In some experiments pH was adjusted to pH 6.5 for these solutions and HEPES was replaced with PIPES. For C. pelagicus, osmolarity was brought to between 1,000 and 1,200 mOsmol kg−1 by adding sorbitol. For HEK 293 cells, the osmolarity was adjusted 290–300 mOsmol kg−1 with glucose. (DOC) [file pbio.1001085.s009.doc]

**Table S2.** Composition of electrophysiology solutions (mM)

|  | ***Coccolithus pelagicus*** | | | | | **HEK 293 cells** | | | | |
| --- | --- | --- | --- | --- | --- | --- | --- | --- | --- | --- |
|  | **E1** | **E2** | **P1a** | **P1b** | **P2** | **E3** | **E4** | **E5** | **P3** | **P4** |
| **NaCl** | 450 |  |  |  |  | 160 |  |  | 30 |  |
| **KCl** | 8 |  |  |  |  | 2 |  |  | 100 |  |
| **MgCl_2_** | 30 |  | 5 | 5 | 5 | 1 | 1 | 1 | 3 | 3 |
| **MgSO_4_** | 16 |  |  |  |  |  |  |  |  |  |
| **CaCl_2_** | 10 | 100 |  |  |  | 1 | 1 | 1 |  |  |
| **NaHCO_3_** | 2 |  |  |  |  |  |  |  |  |  |
| **HEPES** | 20 | 5 | 100 |  | 5 | 100 | 100 |  | 100 | 150 |
| **PIPES** |  |  |  | 1 |  |  |  |  |  |  |
| **MES** |  |  |  |  |  |  |  | 100 |  |  |
| **NMDG** |  |  |  |  |  |  | 75 | 75 |  | 65 |
| **TEA-Cl** |  | 200 |  |  | 200 |  |  |  |  |  |
| **EGTA** |  |  | 5 | 5 | 5 |  |  |  | 1 | 1 |
| **K-Glutamate** |  |  | 200 | 200 |  |  |  |  |  |  |
| **Glucose** |  |  |  |  |  | 20 | 160 | 160 |  | 70 |
| **pH** | 8.0 | 8.0 | 7.5 | 6.5 | 7.5 | 7.8 | 7.8 | 6.5 | 7.0 | 7.0 |
